# Neoadjuvant SBRT combined with immunotherapy in NSCLC: from mechanisms to therapy

**DOI:** 10.3389/fimmu.2023.1213222

**Published:** 2023-08-03

**Authors:** Yanhong Shi, Xiaoyan Ma, Dan He, Bingwei Dong, Tianyun Qiao

**Affiliations:** ^1^ Department of Pathology, Xianyang Central Hospital, Xianyang, China; ^2^ Department of Pathology, Division of Experimental Diagnostic, KingMed Medical Laboratory (Xi’an) Co., Ltd., Xi’an, China; ^3^ Department of Pathology, Xi’an Central Hospital, Xi’an, China; ^4^ Department of Thoracic Surgery, Tangdu Hospital, Fourth Military Medical University, Xi’an, China

**Keywords:** non-small cell cancer (NSCLC), stereotactic body radiation therapy (SBRT), neoadjuvant therapy, immunotherapy, biomarker

## Abstract

The utilisation of neoadjuvant immunotherapy has demonstrated promising preliminary clinical outcomes for early-stage resectable non-small-cell lung cancer (NSCLC). Nevertheless, it is imperative to develop novel neoadjuvant combination therapy regimens incorporating immunotherapy to further enhance the proportion of patients who derive benefit. Recent studies have revealed that stereotactic body radiotherapy (SBRT) not only induces direct tumour cell death but also stimulates local and systemic antitumour immune responses. Numerous clinical trials have incorporated SBRT into immunotherapy for advanced NSCLC, revealing that this combination therapy effectively inhibits local tumour growth while simultaneously activating systemic antitumour immune responses. Consequently, the integration of SBRT with neoadjuvant immunotherapy has emerged as a promising strategy for treating resectable NSCLC, as it can enhance the systemic immune response to eradicate micrometastases and recurrent foci post-resection. This review aims to elucidate the potential mechanism of combination of SBRT and immunotherapy followed by surgery and identify optimal clinical treatment strategies. Initially, we delineate the interplay between SBRT and the local tumour immune microenvironment, as well as the systemic antitumour immune response. We subsequently introduce the preclinical foundation and preliminary clinical trials of neoadjuvant SBRT combined with immunotherapy for treating resectable NSCLC. Finally, we discussed the optimal dosage, schedule, and biomarkers for neoadjuvant combination therapy in its clinical application. In conclusion, the elucidation of potential mechanism of neoadjuvant SBRT combined immunotherapy not only offers a theoretical basis for ongoing clinical trials but also contributes to determining the most efficacious therapy scheme for future clinical application.

## 1 Introduction

Stereotactic body radiation therapy (SBRT) has demonstrated promising outcomes in various solid tumours, particularly in early-stage non-small-cell lung cancer (NSCLC) (1–4). Compared to traditional radiotherapy, SBRT can significantly improve patient prognosis while posing a low risk of toxicity by precisely targeting local tumours and delivering high-dose, hypofractionated therapy (5–8). It is noteworthy that SBRT has a significant advantage over conventional radiotherapy due to its potent immune-activating effect (9). Conventional radiotherapy was previously believed to have immunosuppressive effects, as evidenced by bone marrow myelosuppression and reduced peripheral blood count during treatment (10). This notion was further supported by the use of whole-body irradiation as a myeloablative conditioning before haematopoietic stem cell transplantation (11). However, unlike conventional radiotherapy, the advent of SBRT enables patients to receive higher doses of precise radiotherapy in fewer fractions. The advantage enables SBRT to minimise the potential persistent immunosuppressive effects on the host when compared to conventional radiotherapy (12). In fact, researchers are increasingly recognizing the potent immunomodulatory effects of SBRT, which can convert refractory “cold” tumours into immunotherapy-responsive “hot” tumours (13). For instance, the incorporation of SBRT with immunotherapy in advanced NSCLC patients not only prolonged survival but also significantly increased cytotoxic T cell infiltration within the tumour microenvironment (TME) (14). Given the promising results of combing SBRT and immunotherapy, it is worthwhile to explore whether this approach can be applied to early-stage NSCLC for improved local tumour control and prevention of postoperative recurrence and metastasis.

Clinically, neoadjuvant immunotherapy has demonstrated promising potential in the treatment of early operable NSCLC (15, 16). Unlike traditional neoadjuvant chemotherapy, neoadjuvant immunotherapy not only promotes local tumour control but also activates the systemic antitumour immune response, which is considered a crucial factor in preventing postoperative recurrence and metastasis (17). Numerous clinical trials have confirmed that combining neoadjuvant immunotherapy with chemotherapy can significantly improve the pathological remission rate in patients (16, 18). For example, the CheckMate-816 trial demonstrated that neoadjuvant nivolumab combined with chemotherapy not only significantly increased both pathological complete response (pCR) (24% vs. 2.2%) and event-free survival (EFS) (31.6 months vs. 20.8 months), without increasing the risk of adverse events, compared to neoadjuvant chemotherapy alone (19). Based in these promising clinical trials, neoadjuvant immunotherapy combined with chemotherapy has been approved as the first-line treatment for early operable NSCLC (19). Given this success, it is worthwhile to investigate whether SBRT can also be combined with immunotherapy as neoadjuvant therapy for early operable NSCLC. Indeed, there are ongoing preclinical and clinical trials exploring the potential synergies between SBRT and immunotherapy in the neoadjuvant setting (20–22). However, before conducting further clinical trials and applications, it is important to fully understand the mechanism of interaction between SBRT and antitumour immune response, as well as determine the optimal dosage and scheduling for combination therapy.

Herein, we present an overview of the current status and potential mechanism of neoadjuvant SBRT in combination with immunotherapy, followed by surgery, for the treatment of NSCLC. We also discuss the optimal therapy schedule and predictive biomarkers for clinical application. Furthermore, we highlight future research directions and challenges that require further investigation.

## 2 The interplay between SBRT and antitumour immune response

**Figure 1:**
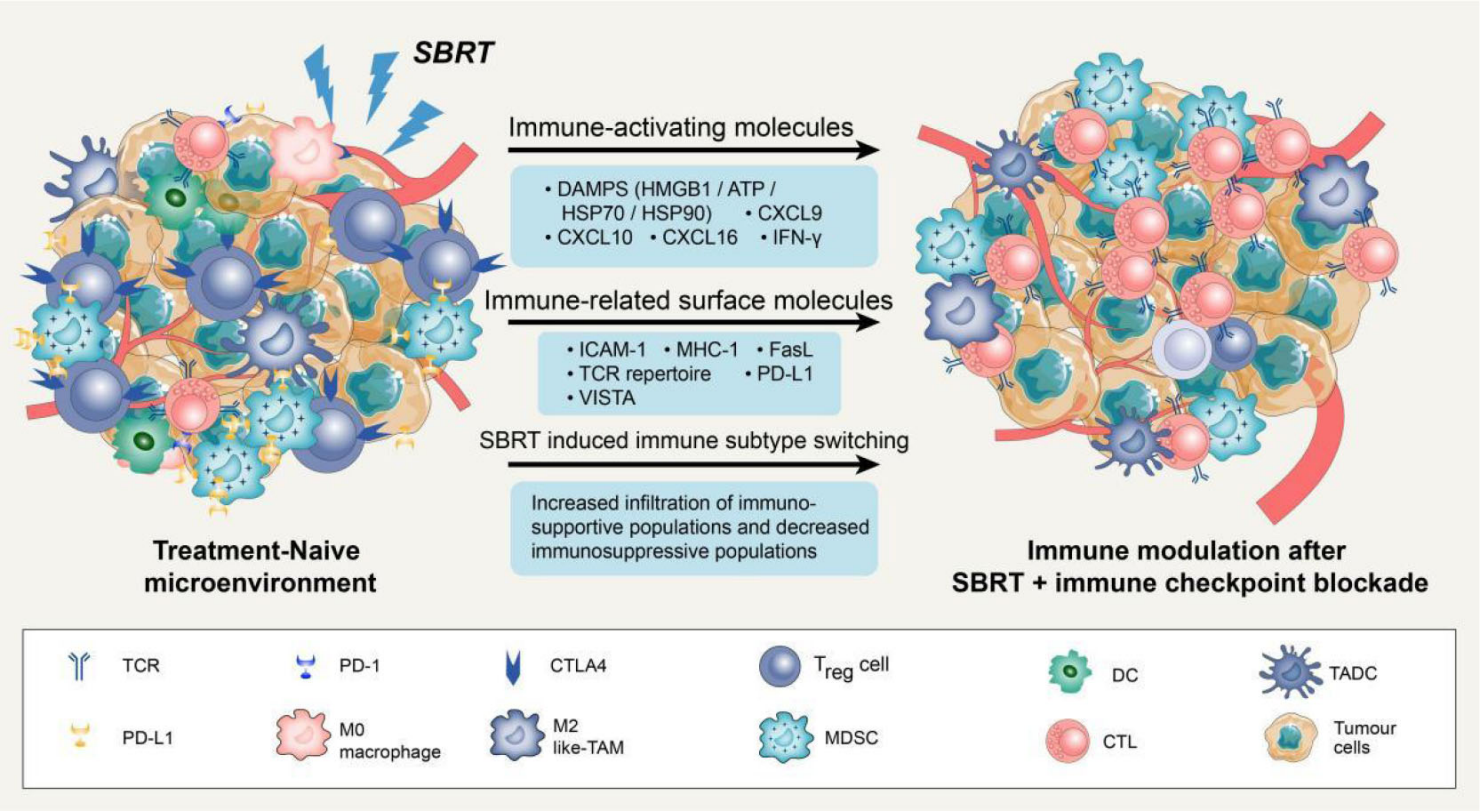
Potential mechanisms of synergy with SBRT and immune checkpoint blockade. Stereotactic body radiation therapy (SBRT) can transfer treatment-naive tumour microenvironment (TME) into “hot” tumour by producing immune-activating molecules and immune-related surface molecules, as well as by directly regulating immune cells composition. Compared with treatment-naive TME, the synergy with SBRT and immune checkpoint blockade could promote the infiltration of immune cells *via* up-regulation of adhesion molecules (ICAM-1) or chemokine (CXCL9/10/16). Notably, SBRT can contribute to adaptive immune resistance *via* IFN-γ mediated up-regulation of PD-L1 and VISTA expression on tumour cells. Blockade of these checkpoints by inhibitors permits the activation of T cells in tumour-associated draining lymph node and TME.

Several studies have suggested that SBRT can promote the antitumour immune response through various pathways beyond its direct DNA damage to tumour cells (23–25). Previous studies have demonstrated that SBRT can induce the presentation of antigens by promoting the release of major histocompatibility complex 1 (MHC-1) and immunogenic cell death (ICD) of tumour cells. Additionally, it can directly stimulate dendritic cell (DC) maturation and CD8^+^ cytotoxic T lymphocyte infiltration in the TME (12, 26, 27). Notably, conventional radiotherapy has been demonstrated to mobilise several immunosuppressive cells, including regulatory T cells (Tregs), M2 macrophages, and bone marrow-derived suppressor cells (28). However, studies on SBRT-related immunosuppressive modification are scarce. A recent study compared the effects of SBRT (40 Gy/3 fractions) with conventional radiotherapy (62 Gy/20 fractions or 66-69 Gy/30 fractions) on the tumour immune microenvironment. The results showed that conventional radiotherapy has a negative impact on systemic immunity, resulting in an increase in neutrophils/lymphocytes and a decrease in total lymphocyte count. In contrast, SBRT increased B cell, central memory T cell, and effector CD8^+^ T cell infiltration in the TME, as well as increased CD8/Treg ratio (29). In summary, SBRT could activate the immune system through multiple pathways and create an ideal TME for subsequent immunotherapy (**Figure 1**).

When tumour cells are exposed to lethal stimuli such as radiation or chemotherapy, a cascade of signaling molecules known as damage-associated molecular patterns (DAMPs) is released (30). These DAMPs include calreticulin, which is exposed to the cell surface, high mobility group box 1 (HMGB1), which is secreted by tumour cells as well as ATP molecules and heat shock proteins (HSP70 and HSP90) (31). Studies have shown that DAMPs induced by ICD could promote cytotoxic T lymphocyte infiltration by facilitating dendritic cell maturation and antigen presentation (32). Moreover, SBRT can stimulate the release of various chemokines such as C-X-C motif chemokine ligand-9/10/16 and interferons (IFNs), which play a crucial role in recruiting activated T cells to infiltrate the TME (33, 34). It has also been reported that SBRT can trigger the exposure and release of numerous tumour-associated antigens, which can be taken up by DCs, transported to lymph nodes, and presented as antigens (35, 36). Overall, these SBRT-induced factors are critical for the activation of local and systemic antitumour immune responses.

SBRT can also enhance the immunogenicity and antigenicity of tumour cells by regulating the expression of cell surface molecules and receptors. For instance, in a dose-dependent manner, SBRT can up-regulate cell surface markers such as intercellular adhesion molecule 1 (ICAM-1), MHC-1, and death receptor Fas (27, 37). It is widely recognized that MHC-1 is an essential co-stimulatory molecule for activating CD8^+^ T cells (38). ICAM-1 is the key adhesion molecule that facilitates immune cell adhesion and migration into the TME (39). The up-regulation of these surface molecules could enhance T cell-mediated antitumour immune response and increase the sensitivity of cytotoxic T lymphocytes to recognize and eliminate tumour cells (40). Notably, SBRT could also increase immune checkpoint expression on the surface of tumour cells. For example, the analysis of paired lung cancer samples following SBRT revealed an increase in the diversity of the T cell receptor repertoire and programmed cell death ligand 1 (PD-L1) expression, while no significant increase of CD8^+^ T cell and IFN expression was observed within tumour tissues (33). In addition to PD-L1, SBRT can also significantly up-regulate V-domain immunoglobulin suppressor of T cell activation (VISTA) expression in CD8^+^ T cells (29). It is worth noting that not all immune checkpoints are elevated following SBRT. Studies have reported that SBRT can significantly increase the frequency of Ki67^+^ programmed cell death protein 1 (PD-1)^+^ T cells and natural killer cells in advanced tumours without a significant increase in immune checkpoints such as T cell immunoglobulin and mucin domain-containing protein 3 (TIM-3) and Lymphocyte activation gene-3 (LAG-3) (41). In conclusion, SBRT-induced up-regulation of certain immune checkpoints might render patients more sensitive to subsequent immune checkpoint inhibitors, resulting in higher response rates and prolonging overall survival (OS).

Previous studies have demonstrated that SBRT can also transfer immunosuppressive microenvironments into “hot” tumors by directly regulating the immune cell composition (42). Reprogramming of the TME after SBRT is primarily induced by the production of chemokines and cytokines to recruit specific immune cell subsets. In mouse tumours, a single high-dose radiotherapy increased the influx of CD8^+^ T cells and simultaneously decreased Treg cell invasion (43). This change may attributed to the release of chemokine and vascular morphological (44). The enhanced homing of immune cells creates an ideal microenvironment for subsequent immunotherapy to effectively elicit antitumour response. A phase 2 clinical trial was conducted to evaluate the efficacy of combing pembrolizumab (a PD-1 inhibitor) with SBRT in NSCLC patients and further studied the reprogramming of TME. Results demonstrated a significant increase in the overall response rate (4.87-fold vs. 2.56-fold) and CD103^+^cytotoxic T cell infiltration after 6 weeks of SBRT plus pembrolizumab therapy, as compared to pembrolizumab monotherapy (14).

In addition to its direct tumour-killing effect, SBRT can induce tumour shrinkage in non-irradiated and distant metastatic tumours through the abscopal effect (45). The current understanding is that local immune activation triggered by SBRT can initiated a systemic immune response that produces cytokines and circulating CD8^+^ T cells. These molecules can then act on distant non-irradiated sites and effectively inhibit metastatic tumour progression (46). While this phenomenon is rare in SBRT monotherapy, combining it with immunotherapy is expected to increase its incidence.

## 3 The preclinical foundation of neoadjuvant SBRT combined with immunotherapy

It is widely acknowledged that neoadjuvant immunotherapy has the potential to not only control local tumours but also inhibit postoperative recurrence and metastasis through systemic immunity (47). While the effects of SBRT on the local TME have been studied, the impact of combining it with immunotherapy on systemic immunity remains unclear. The activation of systemic antitumour immune response is believed to be the mechanism underlying the radiotherapy-induced abscopal effect (48, 49). In a study with mice bearing breast cancer, combining radiotherapy with immunotherapy resulted in significant tumour shrinkage at both irradiated and non-irradiated sites. Notably, the abscopal effect was abolished in T cell-deficient mice (nude mice), indicating that T cells are essential for radiotherapy-induced distal tumour suppression (50).

**Figure 2:**
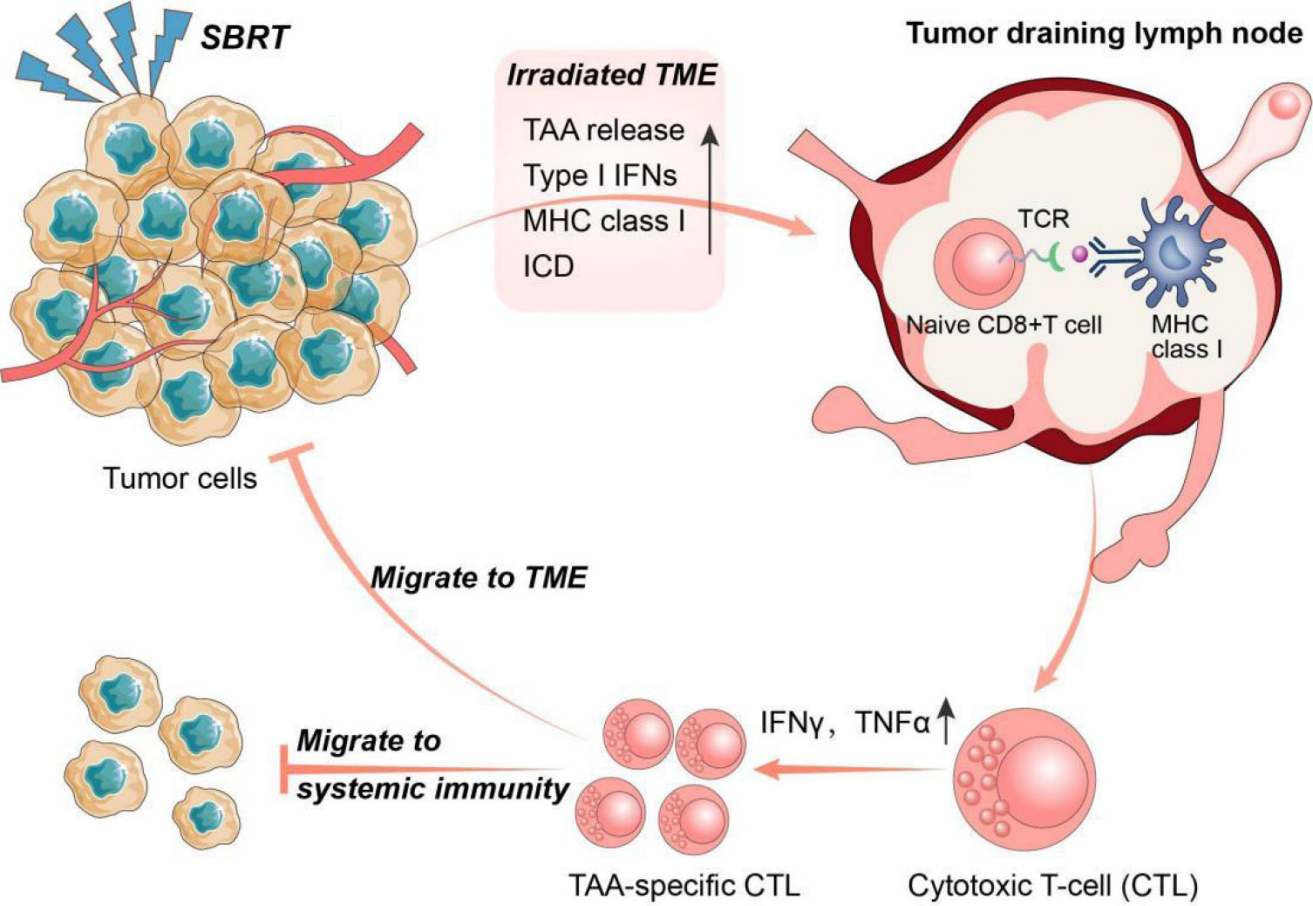
The interplay between SBRT and antitumour immune response. Stereotactic body radiation therapy (SBRT) could promote the activation of anti-tumour immune system through multiple pathways. In turn, activated anti-tumour immune responses also play a key role in the radio-induced abscopal effect. Specifically, SBRT can initiate antigen presentation by promoting the release of tumor-associated antigens (TAA) and major histocompatibility complex I (MHC I), and induce immunogenic cell death (ICD) in tumour cells. Tumour draining lymph nodes (TDLNs) are the main sites of anti-tumour immunity initiation where immune cells develop into progenitor cells after binding to antigens presented by dendritic cells (DCs). The progenitor cells subsequently differentiate into TAA-specific cytotoxic T lymphocytes (CTLs) and migrate into the tumour microenvironment (TME) and systemic immunity.

Tumour-draining lymph nodes (TDLNs) are acknowledged as the primary sites for initiating antitumour immune responses, where immune cells differentiate into progenitor cells upon binding to antigens presented by DCs. These progenitor cells then differentiate and migrate into the TME, contributing to systemic immunity (51). Recently, Huang et al. proposed a novel concept suggesting that the antitumour effects of immune checkpoint inhibitors primarily occur in TDLNs rather than TME. The research found that injecting PD-L1 inhibitors into TDLNs significantly inhibited tumour growth, whereas injecting PD-L1 inhibitors directly into tumours had no effect. Furthermore, surgical removal of TDLNs abrogated the antitumour effects of PD-L1 inhibitors. Further mechanistic studies demonstrated that immune checkpoint inhibitor therapy initially promotes the amplification of T cell factor 1 (TCF-1)^+^ thymocyte selection-associated HMGB (TOX)^-^ CD8^+^ T cells), which are tumour-specific memory cells in TDLNs, These cells subsequently migrated to the TME and peripheral immunity where they differentiate into effector T cells (52). This novel concept highlights the importance of TDLNs and systemic immunity in the antitumour response to immunotherapy (**Figure 2**).

According to research, SBRT has demonstrated a greater potential than conventional radiotherapy in activating immune cells in TDLNs, leading to a more robust systemic immune response capable of eliminating potential metastases. Lee et al. were among the first to report that high-dose radiotherapy (15-25 Gy×1) could enhance the activation of immune cells in TDLNs of advanced tumours, resulting in activated CD8^+^ T cells that not only targeted primary tumours but also eliminated distant metastases in some cases. Moreover, the incorporation of immunotherapy into high-dose radiotherapy resulted in enhanced tumour eradication and systemic antitumour immune response (53). Additionally, Walker et al. demonstrated that the combination of high-dose radiotherapy with bempegaldesleukin (a CD122-preferential interleukin-2 pathway agonist) not only impedes the growth of irradiated tumours but also activated tumour-specific CD8^+^ T cells in systemic immunity, leading to the elimination of non-irradiated metastases (54). Additionally, in a preclinical model featuring disseminated metastasis (4T1 and mouse oral carcinoma 2), researchers discovered that the addition immune checkpoint inhibitors to radiotherapy plus bempegaldesin significantly prolonged the survival of mice by preventing distant metastasis. The effect was achieved by generating immune memory cells in TDLNs (55). Collectively, these preclinical studies suggest that combining SBRT with immunotherapy may enhance the incidence of the abscopal effect by promoting immune cell activation in TDLNs, generating a systemic immune response.

The dose and fraction of SBRT have also been demonstrated to impact the activation of TDLN-mediated systemic immune response. However, there are conflicting data and divergent opinions on the superiority of low-dose SBRT and single high-dose SBRT. Lee et al. discovered that low-dose SBRT (5 Gy×4 over 2 weeks) was significantly less effective in inducing a systemic antitumour immune response than a single dose of SBRT (20 Gy), possibly due to the gradual elimination of effector T cells and subsequent early relapse (53). In contrast, other reports have demonstrated that low-dose SBRT can elicit more favourable local and systemic immune responses and synergize with immunotherapy. For instance, Dewan et al. found that in a bilateral preclinical model of breast cancer, low-dose radiotherapy (8 Gy×3 or 6 Gy×5) combined with immunotherapy was more effective than administering a single high dose of 20 Gy. This combination not only slowed down tumour growth at the irradiation site but also significantly inhibiting lung metastasis and prolonging the survival of mice (56). Furthermore, Schaue et al. demonstrated in a mouse model loaded with B16-OVA melanoma cells that low-dose SBRT (7.5 Gy×2 and 5 Gy×3) was generally superior to a single dose of radiotherapy (15 Gy) in inducing peripheral tumour-specific immune responses (57).

A noteworthy finding from Savage et al. in a preclinical model of lung cancer was the efficacy of a new radiotherapy regimen (22 Gy followed by 0.5 Gy × 4 days) in increasing the infiltration of Granzyme B^+^CD8^+^ T cells within TME, while simultaneously reducing immune suppression caused by Tregs and M2 macrophages when compared to standard SBRT. Further immunoassay of secondary lymphoid organs indicated a significant increase in Granzyme B^+^CD8^+^ T cells and IFN-γ^+^CD8^+^ T cells in TDLNs of mice treated with the new radiotherapy regimen. These promising preclinical results offer a potential new radiotherapy regimen for clinical application to enhance its immunogenic potential (58). Overall, the above preclinical studies provide a foundation for the use of neoadjuvant SBRT combined with immunotherapy in early NSCLC to prevent distant metastasis.

## 4 Advances in neoadjuvant SBRT combined with immunotherapy for NSCLC

The combination of SBRT and immunotherapy has exhibited substantial potential in triggering a systematic antitumour immune response, as evidenced by preclinical studies. Clinically, Shaverdian et al. conducted a prospective analysis and demonstrated that advanced NSCLC patients who received radiotherapy prior to pembrolizumab treatment had significantly longer progression-free survival and overall survival compared to those without previous radiotherapy (59). As for neoadjuvant therapy, preliminarily clinical trials have shown that neoadjuvant SBRT combined with immunotherapy can prolong the survival of patients in several tumours by inducing a systemic antitumour immune response. For instance, in a study of 30 patients with locally advanced oral cavity squamous cell carcinoma, the addition of SBRT to neoadjuvant nivolumab therapy resulted in significant rates of major pathological response (MPR)(60.0%) and pCR(33.3%) in the neoadjuvant therapy group. Additionally, the group receiving combined treatment had an improved 24-month disease-free survival rate (70.4%) and OS rates (76.4%) (60). In another prospective study of locally advanced head and neck squamous cell carcinoma, the combination of neoadjuvant nivolumab and SBRT (40 Gy×5 or 24 Gy×3) significantly improved pathological responses in patients, with 86% achieving MPR and 67% achieving pCR (60).

Several phase II clinical trials have been initiated in resectable early NSCLC to investigate the feasibility, toxicity, and optimal schedule of neoadjuvant SBRT combined with immunotherapy based on increasing preclinical and clinical evidence (**Table 1**). For instance, a multicentre phase II trial (NCT04245514) evaluated the safety and efficacy of adding immunotherapy to neoadjuvant radiotherapy in patients with resectable stage III NSCLC. Durvalumab was administered in combination with SBRT (5 Gy×5 and 8 Gy×3) or conventional radiotherapy (2 Gy×20) to observe 12-month EFS after surgery, with recurrence-free survival and OS as secondary outcomes. Notably, the timing of SBRT initiation in neoadjuvant therapy remains inconsistent across current clinical trials. For instance, in one clinical trial (NCT05319574) for operable stage IB to III NSCLC, SBRT (8 Gy×3) was initiated 1-7 days before the first cycle of immunochemotherapy, whereas another trial (NCT05500092) started SBRT (8 Gy×3) therapy at the end of the first immunotherapy cycle (three cycles of neoadjuvant nivolumab plus chemotherapy). Additionally, in the clinical trial (NCT03110978) conducted by chang et al., nivolumab was administered either 36 hours before or after the initial fraction of SBRT. Significantly, the recent report on this trial demonstrated that combination therapy effectively enhances the 4-year EFS rate to 77%, compared to only 53% (95% CI 42–67%) achieved with SBRT monotherapy (61). The variation in therapy schedules among these clinical trials facilitates the exploration of optimal combination therapy strategies and underscores the need for a thorough understanding of the underlying mechanisms of neoadjuvant combination therapy.

**Table 1:** Clinical trials of neoadjuvant SBRT combined with immunotherapy in NSCLC.

NCT number	Patient tumour stage	Radiotherapy planning	Immunotherapy planning	Primary outcome	Secondary Outcome	Phase
NCT04245514	Resectable Stage III(N2)	Cohort A: 2Gy × 20 weekdaily Cohort B: 5Gy × 5 weekdaily Cohort C: 8Gy × 3 q2d	1 cycle of durvalumab	Event-free survival (EFS) at 12 months	Event-free survival (EFS) Recurrence-free survival (RFS) Overall survival (OS)	2
NCT05319574	Operable stage IB to III	8Gy in 3 daily fractions	2 cycles of tislelizumab (200mg) with platinum-based doublet chemotherapy administered pre-surgery	Major Pathological Response (MPR)	Pathologic Complete response (PCR) Resected rate Disease-free survival	2
NCT05500092	Resectable stage IIA to IIIB	8Gy in 3 daily fractions	3 cycles of neoadjuvant nivolumab and platinum-based doublet chemotherapy	Complete pathological response rate (CPR)	Major Pathological Response (MPR) Event Free Survival (EFS)	2
NCT04933903	Operable stage IB - III	7Gy × 1; 4Gy × 2	Ipilimumab + nivolumab	Number of Patients with a Pathologic Response	Incidence of Treatment-Emergent Adverse Events	2
NCT03217071	Resectable stage I-IIIA	Single 12 Gy	2 cycles of pembrolizumab every 3 weeks	Change in number of infiltrating CD3+ T cells/μm^2^ Proportion of achieving a two-fold increase	Treatment-Related Adverse Events (AEs) Grade 3 immune-related AEs Overall Survival	2
NCT04271384	Stage I	18 Gy × 3 or 10 Gy × 5 or 7.5 Gy × 8	3 cycles of nivolumab every 3 weeks	Pathologic complete response (pCR)	Major pathological response (MPR) Treatment-related adverse events Objective response rate (ORR)	2
NCT03110978	Stage I-IIA or Recurrent	SBRT over 1-2 weeks	3 cycles of nivolumab every 4 weeks	Event-free survival (EFS)	Overall survival (OS) Incidence of treatment-related adverse events	2

## 5 Future challenges and directions for neoadjuvant combination therapy

Further research is needed to determine the optimal dosage, fraction, and schedule of radiotherapy in neoadjuvant combination therapy to enhance the systemic antitumour immune response, which remains a challenging task (62).

Limited research has been conducted on the impact of varying doses and fractions of radiotherapy on local and systemic immune responses in NSCLC patients. A recent clinical trial investigated the safety and efficacy of pembrolizumab combined with SBRT (50 Gy in four fractions) or conventional radiotherapy (45 Gy in 15 fractions) for treating lung and liver metastases in metastatic NSCLC. The results revealed that the group receiving pembrolizumab plus SBRT had an objective response rate of 38%, while only 10% was observed in the group receiving pembrolizumab plus conventional radiotherapy (63). This clinical trial demonstrated the superiority of SBRT over conventional radiotherapy in antitumour metastasis, no differences were observed between different SBRT regimens. Therefore, a convincing preclinical study is required to address this issue. However, the challenge with current preclinical research is the disparity between its findings and applicability in clinical practice (64). The inconsistency maybe attributed to the absence of preclinical models that can accurately replicate the immune microenvironment of patients. Current preclinical models primarily employ murine-derived cell lines in normal mice, and their use of murine-derived immune system and immune checkpoint inhibitors further undermines the reliability of preclinical research outcomes (56). Therefore, the development of a humanised mouse model, in which human immune cells are transplanted into mice with severe combined immunodeficiency, is anticipated to offer a solution to this challenge (65).

In addition to investigating the effects of radiotherapy dosage and fraction on the immune system, determining the optimal schedule is also critical in neoadjuvant therapy. To this end, Dewan et al. conducted a preclinical study using a bilateral breast cancer model to investigate the effect of combination therapy when altering the timing of immunotherapy relative to radiotherapy. The study aimed to investigate the impact of initiating immunotherapy 2 days before, on that same day as, or 2 days after SBRT (8 Gy×3) completion on tumour growth. Results showed that initiating immunotherapy 2 days before or on the same day as radiotherapy ended inhibited tumour growth at both irradiated and non-irradiated sites. However, delaying immunotherapy until 2 days after the completion of radiotherapy reduced therapeutic efficacy, resulting in complete regression of only one of the six primary tumours and reduced growth inhibition in the non-irradiated sites. This indicates that the timing of immunotherapy vs. radiotherapy might influence the efficacy of the combination therapy (56). Similarly, Dovedi et al. investigated the optimal schedule for combining immunotherapy with SBRT (10 Gy×5) by varying the timing of treatment. The results showed that adding immunotherapy at the beginning or end of SBRT did not significantly affect the OS in mice. However, initiating immunotherapy one week after the end of SBRT was entirely ineffective in improving OS, similar to radiotherapy alone. Furthermore, they analysed the dynamics of CD4^+^ T cells and CD8^+^ T cells in the TME to explore the underlying mechanisms of the optimal combination schedule. Results indicated a significant increase in PD-1^+^CD4^+^ and PD-1^+^CD8^+^ T cell proportions within the tumour one day after SBRT completion, but a significant decrease in PD-1^+^CD8^+^ T cells seven days post-radiotherapy (66). Collectively, these preclinical studies suggest that SBRT leads to an acute increase in tumour-specific CD8^+^ T cells. Adding immunotherapy after the completion of the radiotherapy cycle might result in a significant decrease in the treatment’s efficacy due to the anergy of these cells.

Identification of predictive biomarkers for neoadjuvant combination therapy is crucial in determining the population that will benefit and dynamically evaluating therapy efficacy (67). Nevertheless, the biomarkers of neoadjuvant SBRT in combination with immunotherapy remain unknown as classical immunotherapy biomarkers such as PD-L1 and tumour mutation burden fail to dynamically reflect the changes in the TME and systemic antitumour immune response (68). Recent studies have focused on the kinetics of specific immune cell subsets in systemic immunity and their correlation with efficacy (69). For example, Huang et al. reported that TCF-1^+^TOX^-^CD8^+^ T cells in TDLNs are bona fide memory T cells that can migrate and differentiate into systemic immunity after immune checkpoint inhibitor therapy (52). Therefore, the kinetics of CD8^+^ T cell subsets in peripheral immunity after neoadjuvant immunotherapy might be closely associated with efficacy. In addition, Kamphorst et al. reported that increased Ki67^+^PD-1^+^CD8^+^ T cells could be detected in the peripheral blood of approximately 70% of patients with lung cancer 4 weeks after receiving immunotherapy. These cells are considered to be tumour-specific T cells, and their kinetics are correlated with positive clinical outcomes (70). In summary, specific immune subpopulations in systemic immunity might serve as potential biomarkers for dynamically monitoring immune responses in patients with NSCLC undergoing neoadjuvant combination therapy.

## 6 Discussion

Preliminary preclinical findings demonstrate the significant potential of SBRT in combination with immunotherapy in neoadjuvant setting for resectable NSCLC. Furthermore, several ongoing clinical trials are investigating the feasibility and toxicity of this novel neoadjuvant combination therapy; however, it will take some time for data to confirm its clinical efficacy. In addition, the determination of optimal dosage and fractions, identification of predictive biomarkers, and establishment of an optimal schedule for combination therapy are all crucial factors that impact the efficacy of neoadjuvant therapy. Therefore, further preclinical and clinical studies are imperative to address these challenges prior to widespread implementation it in clinical practice.

## Author contributions

YS, XM and DH contribute to the literature research, figures and drafted the manuscript. BD and TQ provided supervision and editing the final manuscript. All authors have agreed to the published version of the manuscript.
